# Multishock Compression Properties of Warm Dense Argon

**DOI:** 10.1038/srep16041

**Published:** 2015-10-30

**Authors:** Jun Zheng, Qifeng Chen, Gu Yunjun, Zhiguo Li, Zhijun Shen

**Affiliations:** 1 Laboratory of Shock Wave and Detonation Physics, Institute of Fluid Physics, P.O. Box 919-102, Mianyang, Sichuan, P. R. China; 2 Laboratory of computational Physics, Institute of Applied Physics and Computational Mathematics, P. O. Box 8009-26, Beijing, P. R. China

## Abstract

Warm dense argon was generated by a shock reverberation technique. The diagnostics of warm dense argon were performed by a multichannel optical pyrometer and a velocity interferometer system. The equations of state in the pressure-density range of 20–150 GPa and 1.9–5.3 g/cm^3^ from the first- to fourth-shock compression were presented. The single-shock temperatures in the range of 17.2–23.4 kK were obtained from the spectral radiance. Experimental results indicates that multiple shock-compression ratio (*η*_i_ = *ρ*_i_/*ρ*_0_) is greatly enhanced from 3.3 to 8.8, where *ρ*_0_ is the initial density of argon and *ρ*_i_ (*i* = 1, 2, 3, 4) is the compressed density from first to fourth shock, respectively. For the relative compression ratio (*η*_i_’ = *ρ*_i_/*ρ*_i-1_), an interesting finding is that a turning point occurs at the second shocked states under the conditions of different experiments, and *η*_i_’ increases with pressure in lower density regime and reversely decreases with pressure in higher density regime. The evolution of the compression ratio is controlled by the excitation of internal degrees of freedom, which increase the compression, and by the interaction effects between particles that reduce it. A temperature-density plot shows that current multishock compression states of argon have distributed into warm dense regime.

The physical properties of warm dense matter (WMD) are a challenging field of research^1^. Generally, WDM covers the states of matter with the density-temperature range of 10^22^–10^25^ cm^−3^ and 0.1–100 eV. In this regime, matter is strongly coupled (1 < *Γ *< 100), mostly degenerate (*Θ* = *k*_B_*T*/*E*_F_ ~ 1), and nonideal^2^. Recently, WDM in the partial ionization regime is also becoming one of important research topics^3^,^4^,^5^,^6^,^7^. The existence of much challenge boosts the development of experiments and theories as the equations of state (EOSs) and the compression technique.

Argon is usually considered as a model substance in WDM region, whose experimental data of EOSs can be used to test and verify the theoretical models. For the purpose, existing experiments reveal that the principal Hugoniot measurements of solid^8^ and liquid^9^,^10^ argon have been performed, and liquid argon has been single-shocked up to the pressure of 91 GPa. The single-shock data were reproduced by a fluid variational theory^11^, which did not include the ionization effect. For the multiple shock compression technique, the obtained data can efficiently extend the applicable range of the models in WDM regime, but it has not been used for argon.

Double-shock data were deduced by the interface continuity condition and strongly depend on the EOSs of the rear anvil, which have been applied in helium^12^, deuterium^13^, and xenon^14^. Several theoretical simulations including QMD^15^, PIMC^15^,^16^, and the chemical pictures^13^,^17^, have been performed, but none of them could give a creditable prediction of the WDM states for different materials. For dense gaseous mixtures of H_2_+He^18^, and H_2_+D_2_^19^, limited third-shock compression data were obtained by the spectral radiance histories of the specimen on the premise of good shock transparency of the anvil interface. Though the shock reverberation technique has been applied to the EOSs study^20^,^21^,^22^, the determination of multiply compressed states were done with the help of hydrodynamic simulations. By now, the integrated multishock compression states diagnostics have not been reported, and shock data of argon above the 100 GPa range are scarce. In addition, no experiment for argon involves in the temperature measurement, which should give an important test for theoretical models.

In our previous work, multiple compression argon^4^ with lower initial densities has been reported. However, it was not integrated especially for the third-shock state and the shock temperature. In this paper, the integrated state diagnostics of argon from single- to fourth-shock compression are developed with an optical pyrometer and a velocity interferometer system. Multishock pressure-density up to 150 GPa and 5.3 g/cm^3^ are obtained. The single-shock temperatures of 17.2–23.4 kK are obtained from the spectral radiance before the loss of the interface transparency appears. Based on these results, we will discuss the evolution of multishock compression properties, and the coupling and the degeneracy parameters of the multishock compression states in the temperature-density plane.

## Results

**Figure 1:**
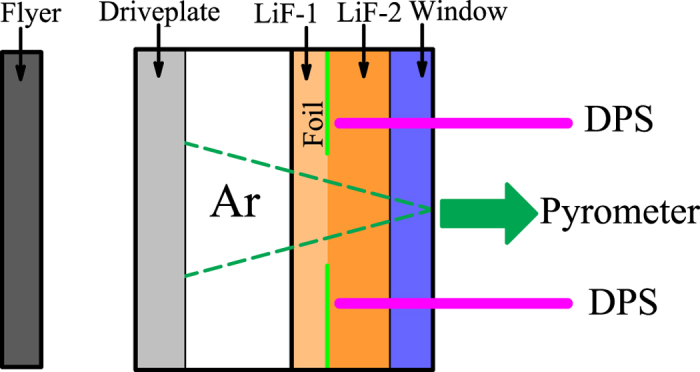
Schematic view of the experimental target and diagnostic devices used to obtain the EOSs of warm dense argon.

**Figure 2:**
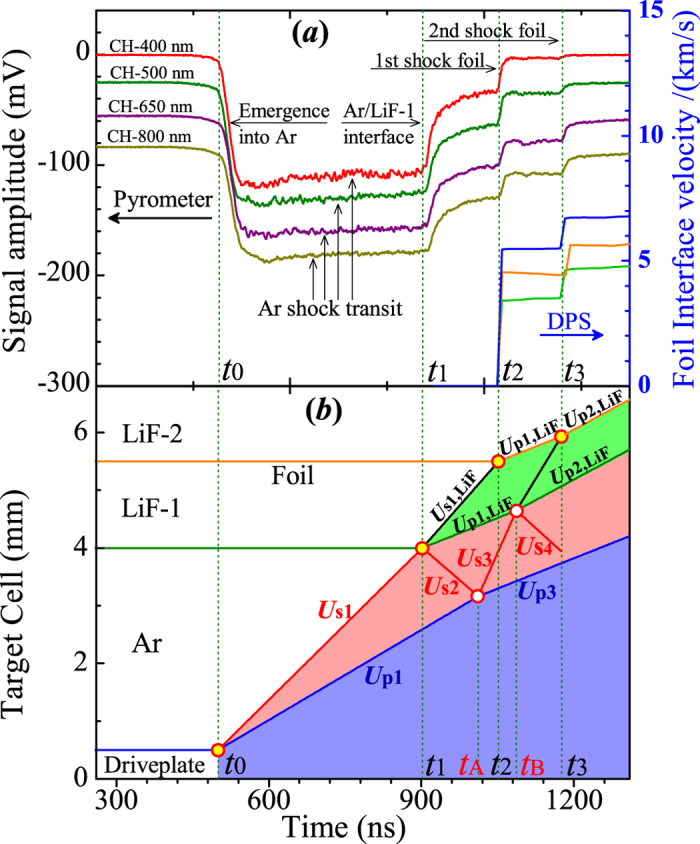
Typical experimental signals and corresponding characteristics for the shot of No. GAr-4. (**a**) self-emission spectral radiance by the pyrometer and interface particle velocity of the foil by DPS. The shock front arrival at the driveplate/Ar interface (*t*_0_), the Ar/LiF-1 interface (*t*_1_), the 1^st^ shock front in LiF-1 arrival at Al foil (*t*_2_), and 2^nd^ shock front in LiF-1 arrival at Al foil (*t*_3_). (**b**) Position-time characteristic diagram showing the trajectories of the shock fronts and interfaces. The corresponding time *t*_0_, *t*_1_, *t*_2_, and *t*_3_ are the same as in (**a**). The time *t*_A_ and *t*_B_ could be deduced by the shock reverberation interaction and discussed in the Sec. III.

The multishock reverberation technique is widely applied to produce WDM. In our experiments, Fig. 1 shows the experimental target and diagnostic devices used to obtain the EOSs of warm dense argon. Before the experiment, the argon specimen was precompressed to about 40 MPa at the surrounding temperature, and the initial density was measured by a method of draining with a special pressure vessel^23^. In the experiment, the representative multishock spectral radiance histories were measured by a pyrometer and the particle velocity profile histories were measured by a Doppler pins system (DPS)^24^, which are shown in Fig. 2(a) for the shot of No. GAr-4. The self-emission spectral radiance provides a clear indication of shock arrival at the corresponding interface of the experimental target cell in Fig. 2(a). Multishock compression states can be determined as follows.

(I) The nearly flat regime (between time *t*_0_ and *t*_1_) indicates that the shock wave propagates steadily through the argon specimen, where warm dense argon with good uniformity is generated. The 1^st^ shock velocity (*U*_s1_) was obtained according to the crossing time and the thickness of the specimen chamber (including the distortion in the precompressed state). The 1^st^ shock particle velocity, pressure, and density (*U*_p1_, *P*_1_, and *ρ*_1_) of argon were determined using an impedance matching method to solve the Rankine-Hugoniot equations^25^. The single-shock temperature (*T*_1_) was obtained from the spectral radiance.

(II) When the shock reached the specimen/LiF-1 interface, the transparency was partially lost due to high shock temperature. As a results, a lower steady signal (between time *t*_1_ and *t*_2_) was recorded. The 1^st^ shock velocity (*U*_s1,LiF_) in the LiF-1 was obtained from the optical radiation signal. The DPS application allowed for direct measurement of the particle velocity of the foil. In fact, the latter almost remained the same as the particle velocity (*U*_p1,LiF_) in the LiF-1 because the shock impedance of the aluminum foil approximately matched with that of the LiF. The 2^nd^ shock particle velocity (*U*_p2_ = *U*_p1,LiF_), the pressure (*P*_2_ = *P*_1,LiF_ = *ρ*_0,LiF_*U*_s1,LiF_*U*_p1,LiF_), and the density (*ρ*_2_) of argon could be deduced by the continuity condition at the interface.

(III) The 4^th^ shock particle velocity of argon was measured by the DPS technique, where the 2^nd^ step signal of DPS expressed the aluminum foil movement (*U*_p2,LiF_) again by the 2^nd^ shock wave in the LiF-1. The detailed trajectories of the shock fronts are shown in Fig. 2(b). The 4^th^ shock particle velocity (*U*_p4_ = *U*_p2,LiF_) and the pressure (*P*_4_ = *P*_2,LiF_) was obtained by the LiF reshock state and continuity condition at interface (the detailed steps described in Ref. 4). With the shock wave propagated into the LiF-2 and the sapphire window, the observed radiance signal gradually became weaker due to the transparency loss of the interface (see time *t*_2_ and *t*_3_ in Fig. 2(a)). Moreover, the 3^rd^ shock states could not be directly determined from the experimental signals, but could be obtained by analyzing the interactions among the waves.

In order to clarify the interaction procedure of the shock waves in detail, the position–time schematic diagram with the driveplate, the specimen, the LiF-1, and the LiF-2 are presented in Fig. 2(b), which shows the trajectories of the shock fronts and the interfaces under multishock compression. The several events of the shock with different interfaces interaction (see time *t*_0_, *t*_1_, *t*_2_, and *t*_3_) are justly corresponding to the experimental signal jump of Fig. 2(a). However, the shock reverberation interactions at the driveplate/Ar interface (time *t*_A_) and Ar/LiF-1 interface (time *t*_B_) cannot be observed. The reverberation time is very important to acquire the 3^rd^ shock state of argon. It can be indirectly deduced by the observed particle velocity, the shock velocity and time points to determine the integrated multishock compression states.

Multiple shock compression is an effective technique to acquire high-pressure state. For the transparent specimen, the states of argon can be analyzed combining the spectral radiance histories and interface particle velocity profile histories in our work. For the opaque or partially transparent argon under multishock compression, the states could be determined by means of the interface time and continuity condition. However, if the time when the shock meets the interface could not be determined rigorously, such as the shock reverberation time at the driveplate/Ar interface, the 3^rd^ shock state determination will become difficult to acquire the whole states from 1^st^ to 4^th^ compression.

The 1^st^, 2^nd^, and 4^th^ shock states determination has been illustrated in section II of this paper. Although the 4^th^ shock pressure and particle velocity have been deduced by the DPS, the integrated 4^th^ shock states including the shock wave velocity (*U*_s4_) and the density (*ρ*_4_) were not obtained. These status messages depend on the third-shock state, which was determined as follows. Firstly, according to the observed 2^nd^ shock particle velocity and the pressure of argon, the 2^nd^ shock wave velocity (*U*_s2_) was acquired by the momentum conservation relation:


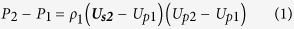


Similarly, the 2^nd^ shock velocity of LiF-1 (*U*_s2,LiF_) was given by


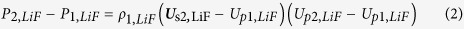


The shock reverberation interaction time at the driveplate/Ar interface (time *t*_A_) and Ar/LiF-1 interface (time *t*_B_) was indirectly obtained by the following method. Based on the shock and reshock wave interactions of argon in the Fig. 2(b), the compression process from time *t*_0_ *t*o time *t*_A_ (in Euler coordinate) was written as


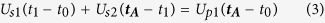


Meanwhile, the shock and reshock interaction processes of LiF-1 from time *t*_1_ to time *t*_3_ in Fig. 2(b) were written as


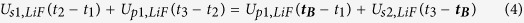


According to Eqs. (3) and (4), the shock reverberation interaction time *t*_A_ and *t*_B_ was acquired. The whole interaction process occurring between the driveplate and LiF-1 anvil was clearly obtained. With the aid of the 2^nd^ and 3^rd^ shock displacement movement of argon in Fig. 2(b), the 3^rd^ shock velocity (*U*_s3_) was determined by


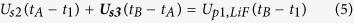


**Figure 3:**
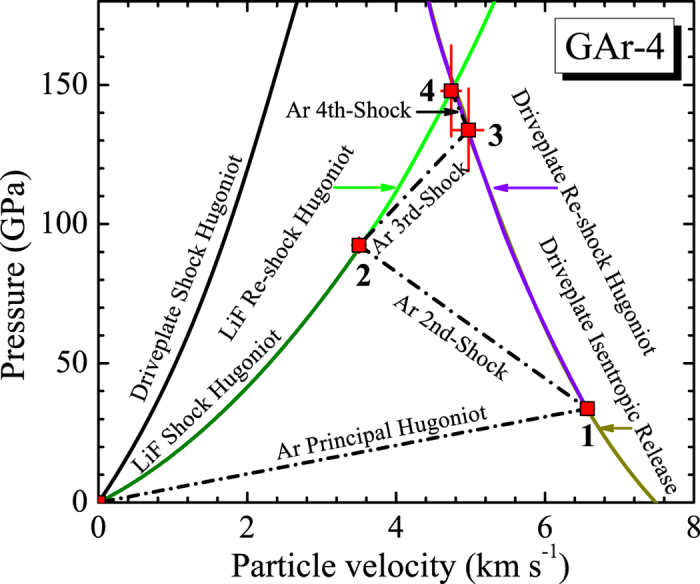
Impedance matching method used to obtain multishock pressure and particle velocity (shot of No. GAr-4). The solid red square symbols indicate the observed states of argon.

The 3^rd^ shock states including the 3^rd^ shock pressure (*P*_3_), the density (*ρ*_3_), and the particle velocity (*U*_p3_) of argon were acquired by the impedance matching method in Fig. 3, and the 4^th^ shock density (*ρ*_4_) was given by


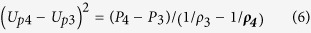


The measured uncertainty for the 3^rd^ shock velocity was determined as follow


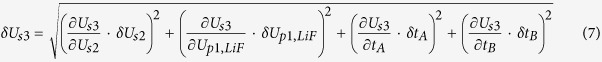


where the uncertainties for the velocity and the time, such as *δU*_s2_, *δU*_p1,LiF_, *δt*_A_, and *δt*_B_ were obtained from Eqs. (1)–(3), 2, 3. Also the 3^rd^ shock pressure, the density, and the particle velocity with the uncertainties could be determined by impedance matching method.

The integrated multishock compression states (from 1^st^ to 4^th^) are listed in Table 1, and the typical results by the impedance matching are shown in Fig. 3. The experimental pressure range of about 150 GPa and the density of about 5.3 g/cm^3^ are presented for warm dense argon, where the 4^th^ shock state approaching to the final equilibrium state can be seen.

Shock temperature data can provide an important validation for the theoretical model. Using the absolute spectral radiance, *I*(λ), of shock front, the single-shock temperature for argon can be extracted from the Planck radiation spectrum:


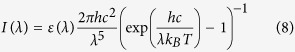


where *T* is the radiation temperature, *c*, *λ*, *h*, and *k*_B_ represent the speed of light, the wavelength, the Planck constant, and the Boltzmann constant, respectively. *ε*(*λ*) is the wavelength-dependent emissivity. In fact, the *ε*(*λ*) depended strongly upon the wavelength, the radiation temperature, the shock front state, and the homogeneity. Since the current measuring range (400–800 nm) is far from the peak wavelength of the Planck distribution, the directly fitted values of the temperature by pyrometry will vary with the variation of the emissivity. Even small errors in the emissivity may greatly influence the fitted temperature values.

**Figure 4:**
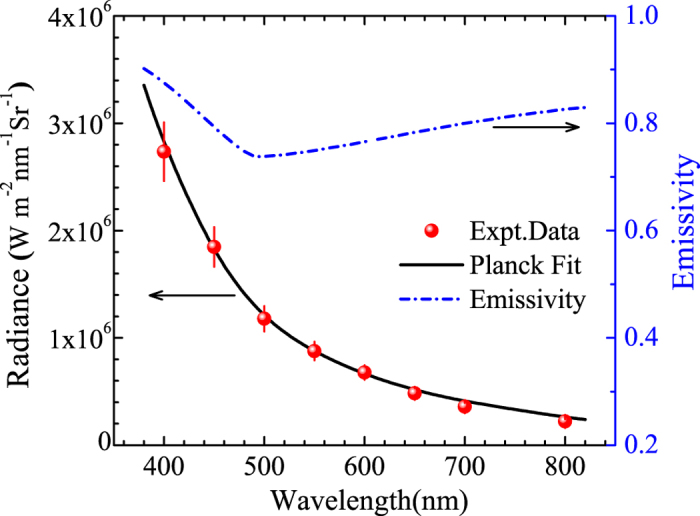
Experimental spectral radiance and the Planck radiation fitting (T_1_ = 23.4 ± 2.6 kK) for shot No. GAr-4. The wavelength-dependent emissivity is calculated and related to the reflectivity by the Drude model.

In current work, *ε*(*λ*) was estimated by the reflectivity of shock front, *R*(*λ*), with the relationship of *ε*(*λ*) = 1–*R*(*λ*). The *R*(*λ*) was related to the complex refractive index of shock front, 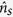, which was calculated by the Drude model for warm dense matter. In the free-electron approximation the complex refractive index is written as^26^,


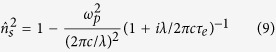


where 
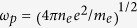
 is the plasma frequency, and 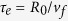 is the electron relaxation time. The parameters of *n*_e_, *m*_e_, and *e* denote the electron density, the electron mass, and the electron charge, respectively. *R*_0_ is the electron mean-free path, which is limited to the interatomic distance. 
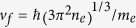
 is the Fermi velocity and *ħ* is the Planck constant. *n*_e_ is necessary to obtain with the calculations. In virtue of Eq. (9), the refractive index 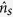 can be obtained. The reflectivity *R*(*λ*) is estimated from the Fresnel’s formula, 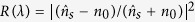, where *n*_0_ is the refractive index in the initial precompressed argon. The emissivity *ε*(*λ*) can be approximately estimated from the *R*(*λ*), and the single-shock temperature is obtained from the nonlinear least squares fit. Figure 4 shows the estimated wavelength-dependent emissivity and the experimental radiance fits for shot of No. GAr-4. Table 1 presents the fitted single-shock temperature values in our work.

## Discussion

**Figure 5:**
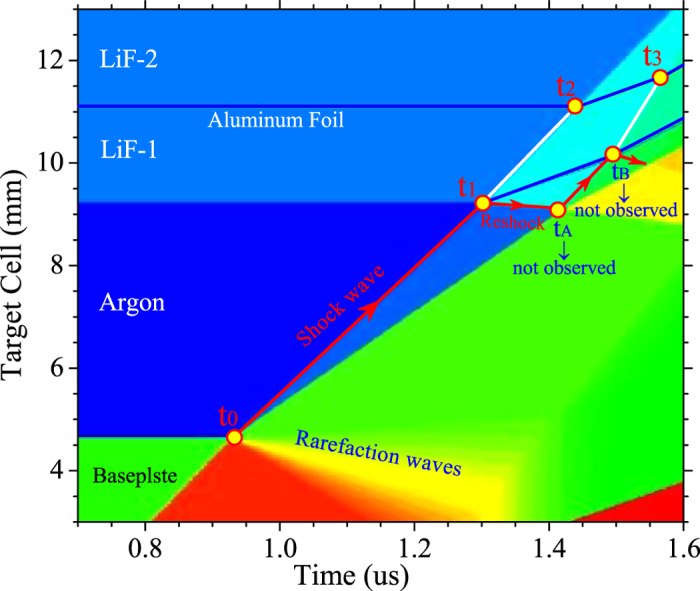
Position-time diagram by one-dimensional hydrodynamic simulation for the trajectories of the shock fronts and interfaces. *t*_0_, *t*_1,_ *t*_2_, and *t*_3_ can be observed by the pyrometer and the DPS. (Symbol meanings as same as Fig. 2).

For the diagnostics of multishock compression states, the target design is crucially important. It is necessary to avoid the influence of rarefaction and catch-up waves on the compressed samples. In our experiments, the wide-thick ratios of the targets were optimized by the calculation of the Lagrangian hydrodynamic equations based on the Riemann solver. Figure 5 shows the calculated trajectories of shock and rarefaction waves and the target interface movement in one-dimensional hydrodynamic simulation for the shot of No. GAr-4. It reveals that the catch-up rarefaction wave makes no influence on both the multishock reverberation diagnostics of dense argon plasma and the shock and reshock compression of LiF-1.

Four experiments were performed with the compressed states probed by a multichannel optical pyrometer and a velocity interferometer system. Table 1 shows the integrated multishock compression states of argon. The states span in the pressure-density ranges of 20–150 GPa and 1.9–5.3 g/cm^3^. The single–shock temperatures determined from the spectral radiance are also listed in Table 1. It is difficult to obtain multishock temperatures from the radiance due to the partial transparency loss of the interface. Hereby, the multishock temperatures are calculated using a model based on the self-consistent fluid variational theory (SFVT)^17^,^27^. In chemical ionization equilibrium, the SFVT model introduced a correlative correction of the ionization energy by fulfilling self-consistency, and considering the interaction between the particles (*i.e*., the atoms, ions, and electrons). This model could reproduce the experimental Hugoniot states of liquid argon^27^, and predict the EOSs and ionization degree of fluid argon. Table 2 shows the calculated results, including the temperature, the ionization degree, the electron density, the coupling and the degeneracy parameters of dense argon plasma. These calculations could be compared with the experiments and characterize the physical properties under multishock compression.

**Figure 6:**
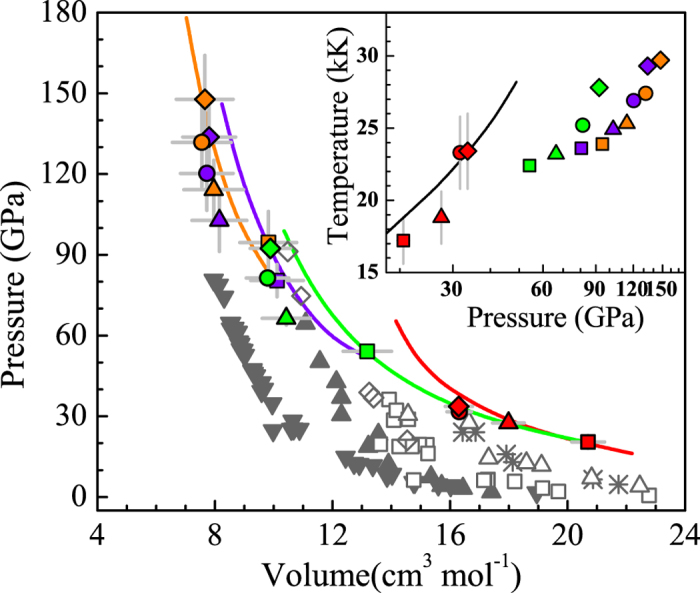
Comparison of argon shock data for different initial states and isotherm data for solid argon. The colored symbols represent the data of our work in the 1^st^ (red), 2^nd^ (green), 3^rd^ (violet), and 4^th^ (orange) shock compression. The solid curves were calculated from the corresponding lowest pressure point. For comparison, the dense argon results (open triangles^28^ and stars^10^), the liquid argon (open diamonds^9^ and open squares^10^), the solid argon Hugoniot (solid triangles^8^) and the solid argon isotherm (inverted solid triangles^29^) are also shown. Inset: The temperatures versus the pressure in our work are represented. The 1^st^ shock data are measured and other data are calculated. The solid curve is the Hugoniot curve given by calculation.

In order to address the thermodynamic properties of multishock argon, the shock pressure versus the volume from 1^st^ to 4^th^ shock compression in this work are shown in Fig. 6, in comparison with previous results whose initial states are argon in the gas^10^,^28^, liquid^9^,^10^, and solid phase^8^,^29^. It is observed that current experimental EOSs results extend the shock pressure to 150 GPa. The molar volume in the range of 7.5–21.0 cm^3^/mol (corresponding to the density of 1.9–5.3 g/cm^3^) was determined by virtue of the multishock reverberation technique. The shock temperatures in the range of 17.2–29.7 kK were also shown as an inset to Fig. 6, with single-shock data determined experimentally and other data given by the model. Fig. 6 also shows the calculated multishock curves approximately reflected from the corresponding lowest experiment points by the SFVT model. It is found that, the calculations are in good agreement with the experimental data within uncertainty of about 13%.

**Figure 7:**
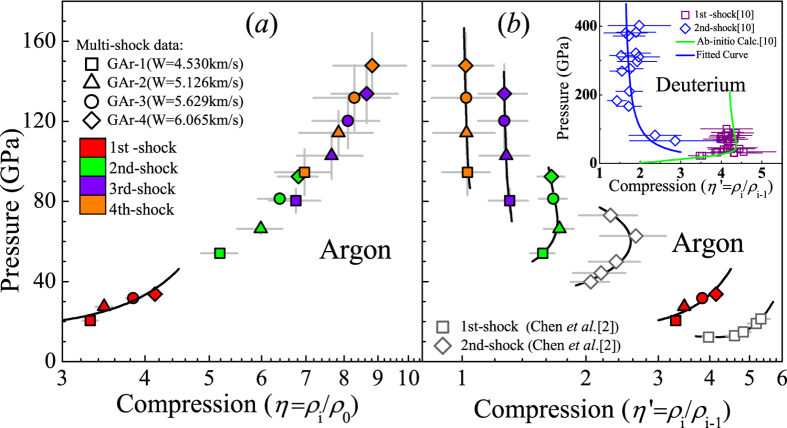
Comparison of multishock compression for gas argon in our present and previous work^4^. (**a**) the compression ratio (*η*_i_ = *ρ*_i_/*ρ*_0_, where i = 1, 2, 3, 4) and (**b**) the relative compression ratio (*η*_i_′ = *ρ*_i_/*ρ*_i-1_, where i = 1, 2, 3, 4) are corresponding with the 1^st^ to 4^th^ shock states, respectively. The present four shots data are described as the solid squares (GAr-1), the solid triangles (GAr-2), the solid circles (GAr-3), and the solid diamonds (GAr-4). Multishock experiments are also shown by the by the 1^st^ (red), 2^nd^ (green), 3^rd^ (violet), and 4^th^ shock (orange) results. The solid curves are fitted from experimental data at the corresponding states. Inset: Single-shock (open squares) and double-shock (open diamonds) compression of liquid deuterium by Z-accelerator^13^ is represented to illustrate the multishock compression.

Until now single-shock compression of WDM has been investigated intensively^8^,^9^,^10^,^11^,^12^. However, multishock compression, especially the change of compression ratios with the compression sequence has less been reported. Figure 7(a) shows the experimental results of argon with the multishock pressure versus the compression ratio (*η*_i_ = *ρ*_i_/*ρ*_0_, where *i* = 1, 2, 3 ,4 is from 1^st^ to 4^th^ shock states, respectively). With the increase of the pressure, the compression ratio increases gradually from 3.3 to 8.8. It indicates that multishock reverberation technique could efficiently enhance the compression.

Figure 7(b) shows the comparison of experimental data of argon between the multishock pressure and the relative compression ratio(*η*_i_′ = *ρ*_i_/*ρ*_i-1_). An interesting phenomena is found in the relationship between the pressure and the relative compression ratio from the 1^st^ to the 4^th^ shocked states for the four groups of experiments. For the 1^st^ shocked states, a higher pressure is associated with a higher relative compression ratio; for the 3^rd^ and 4^th^ compression states, a higher pressure is associated with a lower relative compression ratio. The turning point (corresponding to the maximum relative compression ratio *η*_2_′_max_ = *ρ*_2_/*ρ*_1_ ~ 1.70) occurs at the 2^nd^ shocked states, which shows a non-monotonous relationship between pressure and the relative compression ratio. This phenomenon is more clearly evidenced by our previous experimental results^4^ as is shown by the hollow symbols in Fig. 7(b). A similar phenomenon was also reported for the single and double shock of liquid deuterium^13^ as shown in the inset of Fig. 7. For double shock experiments of deuterium, a higher pressure is associated with a lower relative compression ratio, while for single shock experiments, the relative compression ratio increases with pressure in the low pressure regime and decreases with pressure in the high pressure regime.

In general, the shock compression is controlled by two factors: the internal degree of freedom and the interaction of particles^6^. On the one hand, the total energy of shock wave goes up with the increase of the flyer velocity. It will lead to the enhanced excitation of internal degrees of freedom and the increased ionization in absorbing the thermal energy in the system, which increase the compression. On the other hand, the increase in flyer velocity leads to the increase in the ionization degree and the number density of particles in the system, which could enhance the repulsive interactions between particles and reduce the compression. Therefore, the maximum relative dynamic compression begins to appear at the dominant of atomic excitation in these two effects. Subsequently, with further increase in multishock pressure, a rise number of particles due to the ionization result in the increase in particles repulsive interactions and the decrease in relative compression ratio.

**Figure 8:**
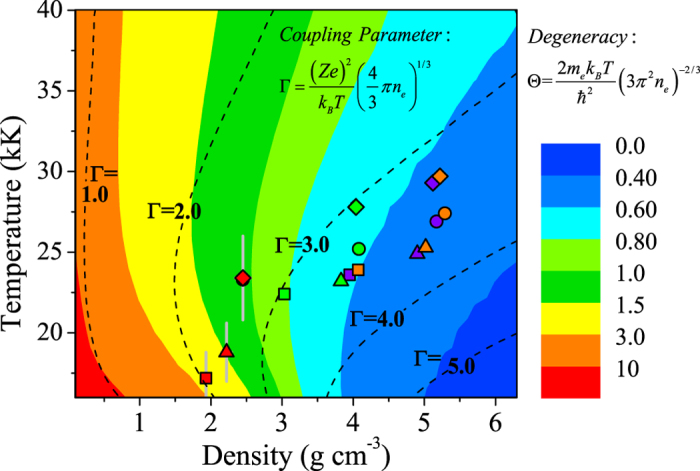
The contours of degenerate (*Θ*) and coupling parameters (*Γ*) of dense argon in the *P-ρ* plane. The current multiple shock compression observed results up to warm dense matter regime are also presented. (Symbol meanings are as same as Fig. 7).

In order to illustrate thermophysical properties of argon plasma under multishock compression, especially the thermal-induced effect in warm dense regime, the coupling parameter (*Γ* ) and the degeneracy parameter (*Θ*) of argon by SFVT calculations are plotted in the temperature-density plane in Fig. 8, as well as the current experimental results. Multishock compressed dense argon plasma with the ionization degree, the coupling parameter, and the degeneracy is presented in Table 2. For multishock states under different flyer impact velocities, the maximum *Γ* and the minimum *Θ* are not appeared at highest pressure regime. As we known, the *Γ is* the ratio of the interaction potential energy to kinetic energy, which reflects on the evolution of potential and kinetic energies in multishock process. In Fig. 8 it can be seen that the *Θ* values are mostly arround 1, and coupled parameter in the range of 2.0 < *Γ* < 4.0. Therefore, the present multishock compression states are mostly degenerate, strongly coupled, and nonideal, which have entered into warm dense regime. These multishock dada are very useful for creating new theoretical models and checking existing ones of warm dense matter.

In summary, warm dense argon was produced via the multishock reverberation technique, and the integrated diagnostics from single-shock to fourth-shock states at the pressure of 150 GPa and the density of 5.3 g/cm^3^ were performed. The current experimental results indicated that the compression could effectively enhanced from 3.3- to 8.8-fold by multiple compression. The limited relative compression ratio *ρ*_2_/*ρ*_1_ ~ 1.70 was observed at double-shock states, subsequently the 3^rd^ and 4^th^ compression slightly decreased with increasing pressure. The evolution of the compression reflects on the interaction effects between particles and on the changes of the excitation of internal degrees of freedom, the ionization, the potential and kinetic energies in multishock-compressed dense argon process. The thermodynamic physical properties of warm dense argon were characterized with the EOSs, compression, coupled, and degenerate parameters. The current diagnostic technique and the calculated model for warm dense argon can be applied to other dense gas, such as rare gases, H_2_, O_2_, and N_2_.

## Methods

The initial precompressed argon was confined in a sandwich assembly including the driveplate, the specimen cell, and the anvils, as schematically shown in Fig. 1. In order to achieve high homogeneity and avoid the influence of rarefaction and catch-up waves, the specimen target design was optimized with the flyer, the driveplate, the sample chamber, and the anvils by the hydrodynamic simulation. By means of the accelerating flyer technique in a two-stage light gas gun, warm dense argon was produced by shock wave. The shock was generated at the interface of the flyer (~3.20 mm thick, tantalum) and the driveplate (~5.00 mm thick, stainless steel 304), and then the shock passed through argon specimen (~4.00 mm thick). The rear of the assembly consisted of two LiF “anvils” and a median aluminum foil. The anvil interface was glued with transparent epoxy Plexiglas. The anvil close to specimen (labeled in LiF-1, ~1.50 mm thick) was used to protect the foil from thermal ablation under shock compression. The other anvil (labeled in LiF-2, ~4.00 mm thick) was plated by the aluminum foil with the front surface outside (~4 mm in diameter central area), which was served as a laser reflective coating in the interface velocity interferometry by the DPS. The sapphire window was employed to prevent the LiF anvils from pre-compressed fracture. Considering the interaction of the waves at the interface, the shock wave in the argon specimen would propagate and reverberate between the driveplate and the LiF-1 with higher shock impedance.

## Additional Information

**How to cite this article**: Zheng, J. *et al*. Multishock Compression Properties of Warm Dense Argon. *Sci. Rep*. **5**, 16041; doi: 10.1038/srep16041 (2015).

## Figures and Tables

**Table 1:** Multishock compression experimental data for dense gaseous argon.

Shot No.	*W* (km/s)	Initial state		1st-shock					2nd-shock				3rd-shock				4th-shock		
		*P*_0_ (MPa)	*ρ*_0_ (g/cm^3^)	*U*_s1_ (km/s)	*U*_p1_ (km/s)	*P*_1_ (GPa)	*ρ*_1_ (g/cm^3^)	*T*_1_ (kK)	*U*_s1,LiF_ (km/s)	**U**_p2_ (km/s)	*P*_2_ (GPa)	*ρ*_2_ (g/cm^3^)	*U*_s3_ (km/s)	*U*_p3_ (km/s)	*P*_3_(GPa)	*ρ*_3_(g/cm^3^)	*U*_p4_ (km/s)	*P*_4_ (GPa)	*ρ*_4_(g/cm^3^)
GAr-1	4.53 ± 0.02	40.20 ± 0.01	0.584 ± 0.001	7.09 ± 0.07	4.94 ± 0.04	20.5 ± 0.2	1.93 ± 0.05	17.2 ± 1.6	8.43 ± 0.25	2.43 ± 0.06	54.1 ± 2.1	3.03 ± 0.19	8.54 ± 1.15	3.86 ± 0.08	80.4 ± 6.0	3.95±0.35	3.54 ± 0.12	94.6 ± 11.5	4.07 ± 0.40
GAr-2	5.13 ± 0.03	40.50 ± 0.01	0.640 ± 0.001	7.76 ± 0.08	5.52 ± 0.04	27.5 ± 0.3	2.22 ± 0.07	18.8 ± 1.8	8.95 ± 0.27	2.81 ± 0.06	66.4 ± 2.5	3.83 ± 0.30	9.42 ± 1.85	4.25 ± 0.14	102.8 ± 11.6	4.90 ± 0.56	4.01 ± 0.08	114.2 ± 10.9	5.02 ± 0.64
GAr-3	5.63 ± 0.03	40.50 ± 0.01	0.638 ± 0.001	8.20 ± 0.05	6.06 ± 0.04	31.7 ± 0.3	2.45 ± 0.06	23.3 ± 2.5	9.60 ± 0.19	3.21 ± 0.09	81.4 ± 2.8	4.08 ± 0.30	9.92 ± 2.08	4.62 ± 0.16	120.2 ± 13.7	5.17 ± 0.60	4.40 ± 0.14	131.8 ± 16.4	5.29 ± 0.70
GAr-4	6.07 ± 0.03	40.05 ± 0.01	0.592 ± 0.001	8.66 ± 0.05	6.57 ± 0.05	33.7 ± 0.3	2.45 ± 0.07	23.4 ± 2.6	10.00 ± 0.14	3.50 ± 0.09	92.4 ± 2.7	4.04 ± 0.28	10.46 ± 2.22	4.97 ± 0.17	133.8 ± 14.8	5.12 ± 0.59	4.74 ± 0.13	147.8 ± 16.3	5.22 ± 0.66

**Table 2:** The calculated dense argon plasma parameters under multi-shock compression. *T, α*, *n*_e_, *Γ*, and *Θ* are the shock temperature, ionization degree, electron density, the coupling parameter, and the degeneracy for argon, respectively. The temperature in a unit is kilo-Kelvin. The electron density in a unit is cm-3. The subscripts1, 2, 3, and 4 meanings are as same as Table. 1.

Shot No.	1st-shock					2nd-shock					3rd-shock					4th-shock				
	*T*_1,cal_	*α*_1_	*n*_e,1_	*Γ*_1_	*Θ*_1_	*T*_2,cal_	*α*_2_	*n*_e,2_	*Γ*_2_	*Θ*_2_	*T*_3,cal_	*α*_3_	*n*_e,3_	*Γ*_3_	*Θ*_3_	*T*_4,cal_	*α*_4_	*n*_e,4_	*Γ*_4_	*Θ*_4_
GAr-1	19.5	7%	0.2 × 10^22^	2.1	2.4	22.4	25%	1.1 × 10^22^	3.0	0.83	23.6	38%	2.3 × 10^22^	3.4	0.62	23.9	47%	2.9 × 10^22^	3.4	0.60
GAr-2	21.2	11%	0.4 × 10^22^	2.5	1.5	23.2	29%	1.7 × 10^22^	3.4	0.64	24.9	43%	3.2 × 10^22^	3.6	0.54	25.3	50%	3.8 × 10^22^	3.6	0.54
GAr-3	22.7	13%	0.5 × 10^22^	2.6	1.1	25.2	34%	2.1 × 10^22^	3.3	0.62	26.9	46%	3.6 × 10^22^	3.5	0.54	27.4	51%	4.1 × 10^22^	3.5	0.54
GAr-4	24.3	16%	0.6 × 10^22^	2.6	1.1	27.8	37%	2.3 × 10^22^	3.0	0.67	29.3	48%	3.7 × 10^22^	3.2	0.56	29.7	51%	4.0 × 10^22^	3.2	0.56
